# Chiral Phonons Enhance Ferromagnetism

**DOI:** 10.1021/acs.jpclett.5c00304

**Published:** 2025-02-18

**Authors:** Jonas Fransson, Yael Kapon, Lilach Brann, Shira Yochelis, Dimitar D. Sasselov, Yossi Paltiel, S. Furkan Ozturk

**Affiliations:** † Department of Physics and Astronomy, Uppsala University, Box 516, 751 21 Uppsala, Sweden; ‡ Department of Applied Physics, The Hebrew University, Jerusalem 9190401, Israel; § Harvard-Smithsonian Center for Astrophysics, Cambridge, Massachusetts 02138, United States; ∥ King’s College, Cambridge CB2 1ST, United Kingdom

## Abstract

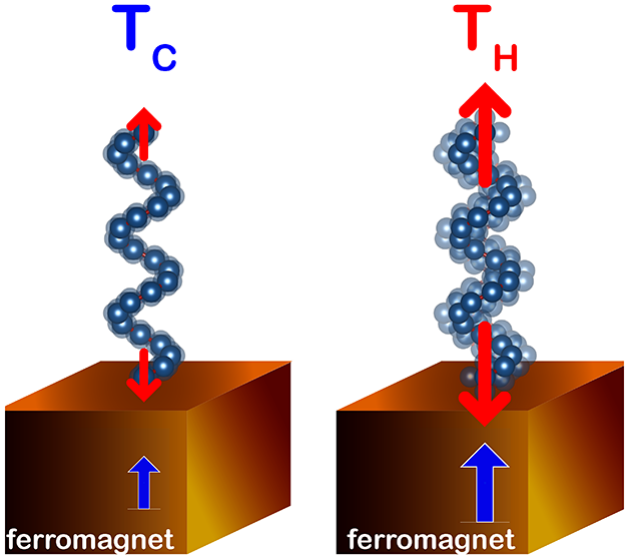

Recent experiments suggest that the conditions for ferromagnetic order in magnetite can be modified by adsorption of chiral molecules. Especially, the coercivity of a ferromagnetic metal was increased by nearly 100% or 20 times the earth magnetic flux density at room temperature. The coercivity was, moreover, demonstrated to increase linearly with the temperature in a finite range around room temperature. On the basis of these results, a mechanism is proposed for providing the necessary enhancement of magnetic anisotropy. It is shown that nuclear vibrations (phonons) coupled to ferromagnetic spin excitations (magnons) absorb the thermal energy in the system, thereby diverting the excess energy that otherwise would excite magnons in the ferromagnet. This energy diversion not only restores the ferromagnetic order but also enhances its stability by increasing the anisotropy energy for magnon excitations. The coupling between phonons with magnons is enabled by chirality due to the lack of inversion symmetry.

Ferromagnetic order typically forms below a critical temperature, *T*_c_, above which higher energy spin excitations become occupied due to thermal fluctuations. Such spin excitations, often referred to as magnons, tend to randomize the order, hence having a detrimental effect on the magnetic state. As the amount of evidence for this behavior that has been collected over the years is overwhelming, the perception of ferromagnetism as a low-temperature collective phase is convincing, albeit that *T*_c_ may be larger than 1000 K in some compounds.^1,2^

Recently, it was reported that supramolecular aggregates may, on the one hand, show stable room-temperature ferromagnetism and, on the other hand, small if not vanishing magnetic moment at a low temperature.^3,4^ These truly surprising results were based on a coercive field, the magnetic field required to flip the magnetization orientation of a compound, which increases with an increasing temperature. When coupling between localized spin moments and phonons is invoked, these results were explained under the condition of broken inversion symmetry such that anharmonic contributions to the phonon excitations become non-negligible.^5^ In fact, breaking inversion symmetry opens for non-vanishing spin-dependent electron–phonon interactions.^6−8^

The phenomenology of magnetism pertains to seemingly separate questions, such as room-temperature ferromagnetism,^9−14^ topological matter,^15−18^ and exotic spin excitations (e.g., skyrmions).^19−22^ Studies suggest that magnetic phenomena are not only pertinent in biological contexts but are also crucial for, e.g., oxygen redox reactions, upon which aerobic life depends.^23,24^ Recently, magnetic phenomena have also been linked to life’s biomolecular homochirality, where the chiral molecular symmetry is broken by processes controlled by electron spin.^25,26^

Nuclear vibrations, phonons, are conventionally considered as a source for decohering and dissipating magnetic states and order. It is, therefore, easy to make the connection between increased temperatures and phonon activation, which, in turn, leads to energy level broadening and occupation of multiple electronic states with competing magnetic properties, ultimately caused by distortions and reformations of nuclear configurations. This faulty conclusion is based on the conjecture that there is an intimate relationship between magnons and phonons. As will be shown in this letter, such a conjecture is not founded on physical grounds but merely presumptions. While nuclear vibrations in a previous publication have been considered as a source for stabilizing magnetic order,^5^ it has previously never been demonstrated that nuclear vibrations provide a sink for the energy that would otherwise induce magnon excitations.

The question to be addressed here is whether nuclear (ionic and molecular) vibrations can increase the magnetic stability in structures that are already ferromagnetically ordered. The investigation is stimulated by the experimental observations on ferromagnetic structures that acquire an increasing coercivity with increasing temperature.^27−29^

In our recent experimental study, we adsorbed chiral molecules onto a ferromagnetic nickel substrate and observed an enhancement in the coercive field of the substrate as the temperature increased.^29^ We provided an explanation for this phenomenon using a thermodynamic model, which we named the chiral heat engine. How can we reconcile the chiral heat engine model, discussed in Kapon et al.,^29^ with the microscopic theory presented here, which involves lattice vibrations?

The chiral-induced spin selectivity (CISS) effect arises from the spin-filtering effects of chiral molecules, which reduce entropy by limiting the available microstates in the electronic spin distribution. In the microcanonical ensemble, the relevant system can be considered the angular momentum distribution, encompassing electronic spin and other angular momentum-bearing degrees of freedom. According to the first law of thermodynamics, the internal energy of an isolated system remains constant; therefore, any work performed by the system must be compensated by heat absorption, as seen in Figure 1. This thermodynamic framework explains the work–heat relationship, but the role of chiral phonons becomes evident when we consider the nature of the heat input. For angular momentum conservation to hold, the heat entering the system must also carry angular momentum. These heat carriers are not merely phonons but specifically chiral phonons that carry angular momentum.

**Figure 1:**
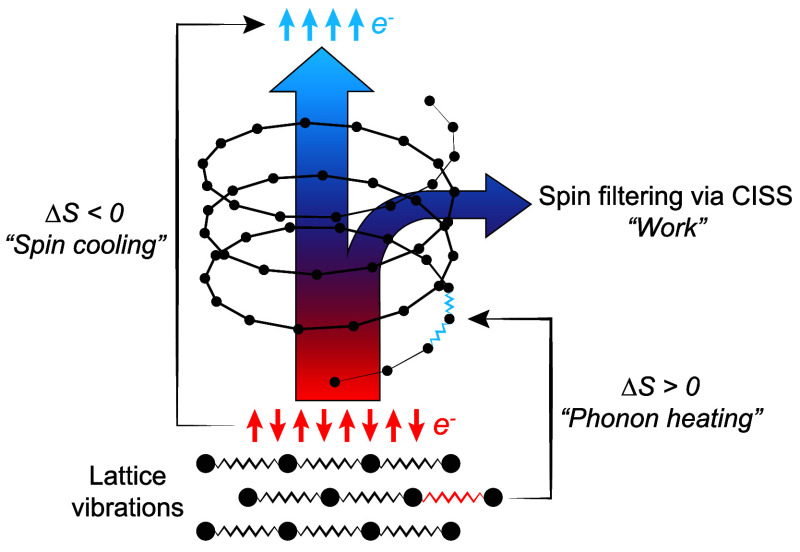
We present an illustration depicting the coupling between chiral phonons and electron spin within a thermodynamic framework. Consider an isolated system of chiral molecules on a ferromagnetic surface, where angular momentum-carrying degrees of freedom are distributed. In a microcanonical ensemble, the system’s total energy remains constant; thus, any work performed by the system must be balanced by heat supplied to it. Through CISS, chiral molecules filter electron spins, reducing the system’s entropy. To maintain energy and angular momentum balance, other degrees of freedom must compensate, with chiral lattice vibrations, phonons, emerging as energetically favorable contributors. Consequently, heating the system to enhance the availability of chiral phonons facilitates the operation of CISS, where the effective cooling by spin filtering is counterbalanced by effective heating from chiral phonons.

However, how do chiral phonons contribute to CISS? In a semiclassical picture, as an electron propagates along a helical path induced by the chiral potential of a closed-shell chiral organic molecule, it experiences an effective Lorentz force. This force redirects the electronic angular momentum, necessitating a corresponding transfer to maintain angular momentum conservation. Closed-shell chiral organic molecules, being non-metallic, lack delocalized, lower energy electrons, which can accommodate this transfer. Thus, the most accessible repository for angular momentum exchange lies in low-energy chiral lattice vibrations. This dynamic interaction between the electron and chiral phonons ensures efficient exchange of angular momentum in a closed system, enabling the electron to maintain its helical trajectory. Thus, chiral phonons play a critical role in facilitating angular momentum conservation while the electronic spin distribution is filtered, effectively fueling the chiral engine.

The model presented in this letter is aimed at capturing the ferromagnetic properties of the surface magnetism of, e.g., magnetite (Fe_3_O_4_) or Ni, onto which a layer of chiral molecules is adsorbed, as experimentally investigated by Ozturk et al.^30^ and Kapon et al.^29^ Specifically, the increased coercive field that results from the presence of the molecules as well as its further increase with the temperature is the target here. Pertaining to magnetite and other local moment ferromagnets, the model is based on localized spin moments as well as nuclear motion, where both quantities are embedded in an electronic structure that constitutes the spin–spin and spin–vibration couplings, discussed in detail in ref (31). As a model for a ferromagnetic lattice, such a model pertains very well to magnetite due to the local moment structure. Its relevance to Ni crystals is reasonable, despite the lack of well-defined localized moments in Ni crystals in comparison to magnetite.

In compounds like magnetite, ferromagnetism is a consequence of a long-range order among the localized spin moments carried by Fe, where the presence of oxygen provides the ferromagnetic exchange interaction through double exchange. Local moment ferromagnets can be effectively modeled using the anisotropic Heisenberg model , where **M**_*m*_ defines the magnetic moment associated with the spatial coordinate **r**_*m*_, subject to isotropic, *J*_*mn*_, and anisotropic, *I*_*mn*_, interactions with the magnetic moment **M**_*n*_ at **r**_*n*_.

For two-dimensional (2D) structures, pertaining to the present context, this model enables an out-of-plane ferromagnetic (*J*_*mn*_ > 0) ground state as long as the anisotropic interaction *I*_*mn*_ is negative. The anisotropic interaction provides an energy gap in the excitation spectrum (Figure 2a), which may be overcome by, for instance, a thermal supply of energy.

**Figure 2:**
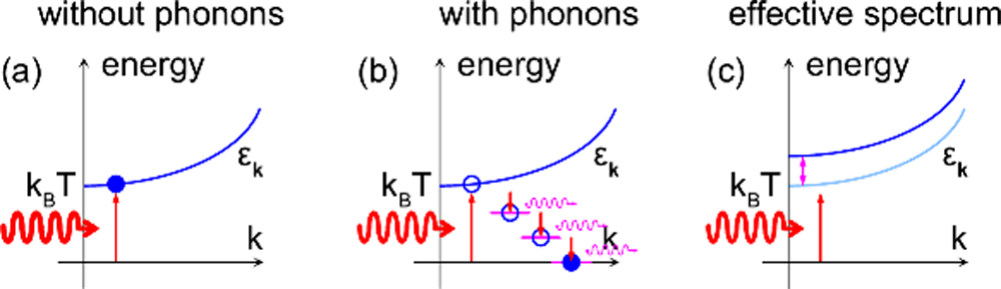
Magnon spectrum (a) without and (b) with coupling to chiral phonons and (c) effective magnon spectrum caused by the magnon–phonon interactions. Without magnon–phonon coupling (a), the thermal energy from the environment (red wiggle) leads to magnon excitations (blue circle). With magnon–phonon coupling (b), the magnon excitation repeatedly loses energy to the phonons (magenta wiggles), successively decaying back to the ground state by occupying intermediate energy levels. Effectively, (c) phonon-induced decay leads to an increased anisotropy.

Here, we consider a novel mechanism that provides an additional source of anisotropy to the magnetic structure. Quite surprisingly, this mechanism is connected to lattice vibrations, phonons (Figure 2b), which absorb the thermally supplied energy such that the magnetic excitations remain unoccupied and, thereby, stabilize the ordered state. Effectively, the magnon–phonon interactions lead to increased magnetic anisotropy (Figure 2c). A requirement for phonons to couple to the spin degrees of freedom is broken inversion symmetry.^6^ In the present context, the broken inversion symmetry is granted by a layer of chiral molecules adsorbed on the surface of the ferromagnet, thereby introducing a coupling between the phonons and local spin moments. Other mechanisms for couplings between spins and the lattice motion have been considered in, for instance, ref (31).

We illustrate the origin of the magnon–phonon interactions in Figure 3, which arises from a phonon-assisted spin–orbit coupling discussed below (eq 2). This phonon-assisted mechanism yields a net contribution in chiral structures because of broken inversion symmetry. A scattering in one direction is not immediately compensated by an equally large scattering in the opposite direction, which leads to a non-vanishing net contribution. In inversion symmetric achiral structures, on the other hand, any scattering is always compensated by an equally large scattering in the opposite direction. The phonon-assisted spin–orbit coupling is, hence, suppressed in achiral structures.

**Figure 3:**
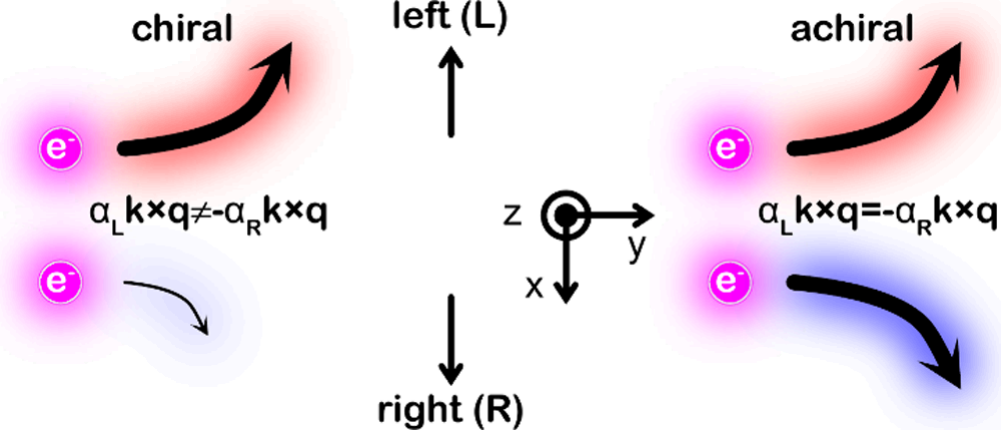
Schematic of the phonon-assisted spin–orbit coupling in (left) chiral and (right) achiral structures. The broken inversion symmetry in the chiral structure leads to the effects of scattering; e.g., left and right, not canceling. Hence, there is non-vanishing net spin–orbit coupling remaining. In the achiral (inversion symmetric) structure, the effects of scattering left and right are equally large and with opposite signs such that their net contribution cancels.

The layer of chiral molecules is assumed to provide phonons through the vibrational normal modes, **Q**_*m*_ = *l*_*m*_***ε***_*m*_*Q*_*m*_, of the nuclear displacement. Here, the operator *Q*_*m*_ = *b*_*m*_ + *b*_*m*_^†^ represents the quantized nuclear displacement, where *b*_*m*_ (*b*_*m*_^†^) is the phonon annihilation (creation) operator, whereas *l*_*m*_ defines a length scale and ***ε***_*m*_ denotes the phonon polarization vector. The phonon background is modeled by the harmonic chiral phonons , where ω_*m*_ is associated vibrational energy. The spin and displacement variables are coupled through the electronic structure in the contribution , where  defines a coupling tensor between the magnetic and mechanical degrees of freedom. Making use of the notation for the nuclear displacement, we set . Then, the model can be summarized as
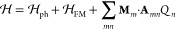
1

The origin of the coupling tensor  is local spin-exchange and electron–phonon interactions between the valence electrons and the localized magnetic moment **M**_*m*_ and nuclear displacement **Q**_*n*_, respectively.^31^ Due to non-locality of the electronic structure, the local interactions are coupled through the associated spin-charge susceptibility, which is non-trivial if either the (i) time-reversal symmetry or (ii) inversion symmetry is broken.

Concerning the inversion symmetry, we make the following further observations. The local electron–phonon coupling originates from the electronic confinement potential *V*(**r**) = ∫*V*(**r**, **R**)d**R** as an effect of the expansion **V**(**r**) = ∫[*V*(**r**, **R**_0_) + **Q**·∇*V*(**r**, **R**_0_) + ...]d**R**_0_, where **R**_0_ refers to integration over the nuclear equilibrium configuration and **Q** = **R** – **R**_0_ represents the nuclear displacement. Specifically, the spin–orbit coupling ∼∇*V*(**r**) × **p** gives rise to a spin-dependent electron–phonon coupling.^6^ Making a plane wave expansion of both the electron and phonon degrees of freedom leads to the phonon-induced spin–orbit coupling being written in terms of the Hamiltonian
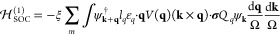
2where the notation *q* = (*m*, **q**) includes the phonon mode *m* and wave vector **q**, ξ = 1/4*c*^2^ is in atomic units, *l*_*q*_ defines a length scale in terms of the phonon density at the frequency ω_*q*_ confined in the volume Ω, and ***ε***_*q*_ denotes the phonon polarization vector. Written like this, it becomes clear that, for an inversion symmetric system, the integration over **q** averages to 0, because the integrand is odd as a function of **q**. Only in a setup with broken inversion symmetry, phonons may contribute to the effective spin–orbit coupling. In the following discussion, we shall not make use of the expansion in wave vector **q** and simply refer to the vibrational normal modes *m*.

The result briefly surveyed above can be understood as an effect of anharmonicity due to broken inversion symmetry. In any setup, the nuclear vibrations can be decomposed into harmonic and anharmonic modes. While harmonic modes may be defined for any type of structure, anharmonic modes always have fully compensating modes in inversion symmetric structures. Whenever the inversion symmetry is broken, however, not all anharmonic modes have fully compensating modes, resulting in a net contribution to the phonon-induced spin–orbit coupling. We notice that, because inversion symmetry is broken in a chiral structure, the necessary conditions for the phonon-assisted spin–orbit coupling to be present are fulfilled. Simply stated, the phonon-assisted spin–orbit coupling is non-zero for chiral molecules and alternates in sign with the enantiomers.

Deviations from the ferromagnetic ground state can be effectively studied through the magnon spectrum. It is particularly interesting to consider the magnon dispersion and whether the interactions with the phonons can introduce an anisotropy that adds to the immanent anisotropy *I*_0_. The reason is that this would give an answer to whether the phonons may increase the coercivity of the magnetic state. To this end, the Holstein–Primakoff transformation^32^ is applied, with the longitudinal magnetic moment *M*_*m*_^*z*^ = *M* – *n*_*m*_ represented in terms of the deviations *n*_*m*_ = *a*_*m*_^†^*a*_*m*_ from the ground-state moment *M*. In this scheme, the Bosonic ladder operators for increasing and decreasing the magnetic moment is defined by  and , respectively, in terms of the magnon annihilation (*a*_*m*_) and creation (*a*_*m*_^†^) operators. The Holstein–Primakoff transformation is useful for magnetic moments *M* > ^1^/_2_, thanks to the enhanced convergence rate of the expansions of the square roots in the expressions for *M*_*m*_^±^. In this sense, the transformation pertains to magnetite where *M* = ^3^/_2_.

By expansion of the model  to quadratic order in the magnon operators, an approximate magnon–phonon model can be established as
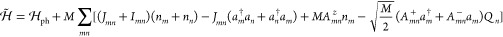
3This expression is obtained by retaining magnon operators up to quadratic order and introducing the notation *A*_*mn*_^±^ = *A*_*mn*_^*x*^ ± *iA*_*mn*_^*y*^.

The interaction parameters *J*_*mn*_, *I*_*mn*_, and  are assumed to all be distance-dependent on the form, e.g., *J*_*mn*_ = *J*(**r**_*m*_ – **r**_*n*_). This enables the plane wave expansion  and analogously for *I*_*mn*_ and **A**_*mn*_. Expanding the magnon operators in corresponding plane wave operators, that is, , the model can be written
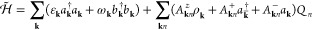
4where ρ_**k**_ = ∑_**q**_*a*_**k**+**q**_^†^*a*_**q**_ is the magnon density operator and  is the magnon energy dispersion in the absence of phonons, whereas  with  or *I*. Moreover, the magnon–phonon couplings 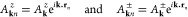 where 
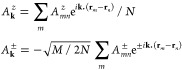
 respectively, and **k̅** = −**k**.

We consider the magnon properties through the Green function . Expanding this propagator order by order in the interaction parameter *A*_**k***n*_ to the lowest non-trivial order, we obtain the correction
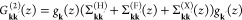
5where *g*_**k**_(*z*) = 1/(*z* – ε_**k**_) is the unperturbed Green functions, whereas the self-energies are given by
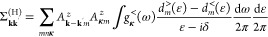
6a
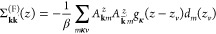
6b
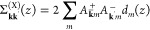
6cThese self-energies are depicted in Figure 4, showing (a) the Hartree Σ^(H)^, (b) the Fock or exchange loop Σ^(F)^, and (c) the lowering–raising Σ^(X)^ diagrams. The arrowed lines and wiggles depict the magnon (*g*_**k**_) and phonon (*d*_*m*_) Green function, respectively. The notation used for these self-energies is defined in the following.

**Figure 4:**
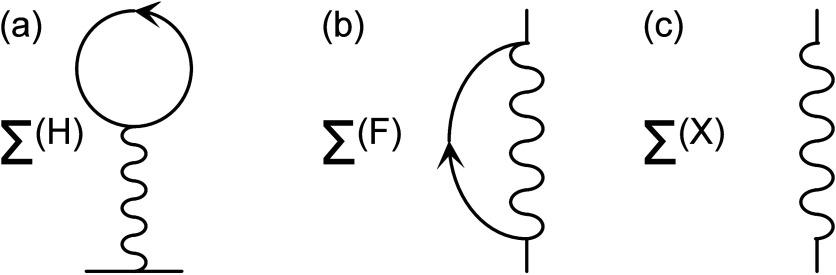
Second-order self-energy diagrams: (a) Hartree, (b) Fock or exchange loop, and (c) lowering–raising diagram. Lines with arrows and wiggles represent magnon and phonon propagations, respectively.

Physically, the diagrams, shown in Figure 4, indicate phonon coupling between magnons at different levels. The simplest process is provided in the lowering–raising self-energy Σ^(X)^, in which there is an angular momentum exchange between the magnons and phonons, indicated by the raising and lowering transfer rates *A*_**k***m*_^±^. For a second order, processes of this type contribute an energy shift to the magnetic anisotropy as well as broadening, which indicates dissipation. The plots in Figure 5a show an example of the real and imaginary parts of the lowering–raising self-energy. For low energies, this self-energy decreases the magnetic anisotropy, while it is increased at high energies. Simultaneously, the line broadening introduced by the angular momentum transfer via phonons is nearly constant throughout the bandwidth of the phonon spectrum. In the calculations, we have used 500 vibrational modes in the range of *J*/30 ≤ ω_*m*_ ≤ 50*J*/3, with couplings *A*_**k**_^±^ = *J*/500 and *A*_**k**_^*z*^ = 3*J*·10^–6^ and Γ_ph_ = 3*J*/100 for *J* = 30 meV.

**Figure 5:**
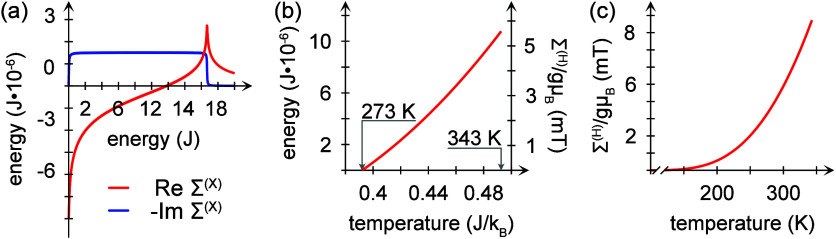
Examples of the self-energies (a) Σ^(X)^ and (b) Σ^(H)^. Here, in panel a, *A*_**k**_^±^/*J* = 1/500, and in panels b and c, *A*_**k**_^*z*^ = 3*J*·10^–6^ and summing over 500 vibrational modes in the range of 1/30 ≤ ω_*m*_/*J* ≤ 50/3. In panel a, the broadening Γ_ph_ = 3*J*/100 has been included for the sake of smoothening the lines. The plots in panels b and c have been subtraced by Σ^(X)^(ω = 0).

The Hartree contribution Σ^(H)^ accounts for a simple phonon-mediated magnon–magnon interaction, where the lesser magnon Green function *g*_**q**_^<^ is proportional to the magnon occupation at the energy ε_**q**_, whereas the difference between the greater and lesser phonon Green functions, *d*_*m*_^>^ – *d*_*m*_^<^, accounts for the density of phonon states at the energy ω_*m*_. This self-energy is evaluated by assuming a 2D structure, corresponding to the interface between the ferromagnet and the chiral molecules. Near the Γ point, the energy dispersion *J*_**p**_ ≈ *J*_0_ + α*p*^2^, where . Using *g*_**q**_^<^(*t*, *t*) = (−*i*)*n*_B_(ε_**q**_), where *n*_B_(ω) is the Bose–Einstein distribution function, and *d*_*m*_^>^(ε) – *d*_*m*_^<^(ε) = (−*i*)2π[δ(ε – ω_*m*_) – δ(ε + ω_*m*_)], the Hartree contribution becomes
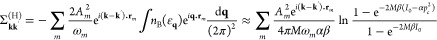
7where e^*i***q**·**r**^ is replaced by 1 and *p*_c_ is an upper cutoff for the quadratic dispersion. For ferromagnetic exchange, α > 0, this contribution is always positive because *I*_0_ – α*p*_c_^2^ < *I*_0_.

With this contribution, the magnon energy is given by
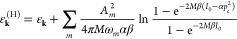
8Effectively then, the total out-of-plane anisotropy  is shifted by the amount represented by the Hartree self-energy. This can be viewed in panels b and c of Figure 5, showing a monotonic increase of the additional energy, as a function of the temperature. In Figure 5b, the plot is limited to a range between 273 and 343 K, which is a viable range in a biological context. Here, the anisotropy increases nearly linearly with the temperature. The properties of Σ^(H)^ for a wider temperature range are shown in Figure 5c. In both of these plots, the value Σ^(X)^(ω = 0) has been subtracted.

The exchange loop, Σ^(F)^, modifies the magnon spectrum by introducing resonances at energies ε_**q**_ ± ω_*m*_, corresponding to energy exchanges between the magnon and phonon subsystems. On the other hand, this contribution is pinned to the phonon energies and does not add substantially to the magnetic anisotropy, in comparison to the Hartree contribution. Therefore, in the following discussion, this contribution will be omitted.

Magnons are excitations from the ferromagnetic ground state of the crystal. Hence, exciting magnons deteriorate the ferromagnetic order, which, in turn, is a signature of weakened ferromagnetism. The magnetic anisotropy, on the other hand, defines an energy scale that must be overcome for the magnons to begin to become populated. In other words, the magnetic anisotropy provides stability to the magnetic order, which can only be overturned by feeding energy into the crystal. When the temperature is raised, magnons may become thermally excited when the energy associated with the temperature is at least as large as the anisotropy. However, if instead the thermal energy can be channeled into excitations that are not detrimental for the magnetic order, the stability of the magnetic state remains. Here, it has been shown that phonons that are coupled to the magnons absorb the thermal energy such that the magnon excitations are suppressed. In addition, the higher the temperature, the more phonons are excited, which adds to the magnetic anisotropy such that the magnons become even less available, hence strengthening the ferromagnetic order.

It should be remarked that the coercivity of the structure depends upon not only the magnon gap but also other mechanisms; for example, multi-domain structures and charge transfer between the molecules and the magnets may influence the total coercive properties. On the other hand, while these and other effects may be important for a complete description, they are unlikely to vary strongly with the temperature and enhance the coercive field in the way that is captured by the influence of the chiral phonons. A study of the impact of other mechanisms is, however, beyond the scope of the current letter.

To construct a deeper comprehension of the relation between the magnons and phonons, we consider the entropy of production flux in the system. In particular, we are interested in whether the entropy of the magnetic subsystem is lowered as a result of the phonon-induced magnon gap. The purpose is accordingly to evaluate the entropy production rate for the magnons related to the Hartree approximation used above in the discussion of the vibrational contribution to the magnetic anisotropy. The entropy production rate ∂_*t*_*S* is provided by the relation , for a zero chemical potential. To relate the maintained ferromagnetic order to losses, the relevant quantity to calculate is the rate of change of the internal magnon energy, ∂_*t*_∑_**k**_⟨ε_**k**_*a*_**k**_^†^*a*_**k**_⟩, because this reflects the gain or loss of entropy due to the coupling to the phonons. Under the assumption that the magnon energy ε_**k**_ is time-independent, this rate of change is given by
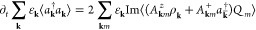
9

The second contribution, , is conservative to the lowest order and reflects the exchange of energy back and forth between the magnonic and phononic subsystems. In contrast, the first contribution comprises an entropic loss, which to the lowest order in the magnon–phonon interactions is given by
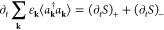
10where  denotes the magnonic entropy gain (+) or loss (−) from the magnon–phonon interactions and
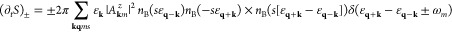
11for *s* = ±1. The physics contained in eqs 10 and 11 is a manifestation of phonon-assisted transitions between different magnon states. The term in this expression with *s* = −1, *n*_B_(−ε_**q**–**k**_)*n*_B_(ε_**q**+**k**_)*n*_B_(ε_**q**–**k**_ – ε_**q**+**k**_), reflects the lowering of the magnon energy (and momentum) through the transition |**q** + **k**⟩ → |**q** – **k**⟩ upon emission of the energy quanta ε_**q**–**k**_ – ε_**q**+**k**_. This energy quantum is subsequently absorbed by the phonon reservoir. The second term (*s* = 1) reflects the opposite type of processes. Finally, the presence of the factor with delta functions in eq 11 emphasizes the occurrence of both absorption, ε_**q**+**k**_ – ε_**q**–**k**_ – ω_*m*_, and emission, ε_**q**+**k**_ – ε_**q**–**k**_ + ω_*m*_, of magnon energy due to the magnon–phonon interactions. The overall sign of the expression in eq 10 indicates whether the magnons lose (−) or gain (+) entropy in this connection.

The continuous lowering of the energy of the magnon population by disposing it to the phonon reservoir leads to the ferromagnetic order being maintained and, with increasing temperature, also further stabilized. It may be noticed that  because the summand expressed in eq 11 is positive. Furthermore, by integration of the angular dependence, the entropy production rate can be written as
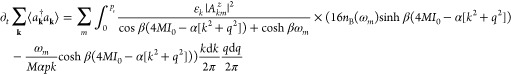
12Both terms in the integrand are negative for a ferromagnet with *I*_0_ < 0. From this observation, we can conclude that the entropy production rate is negative. Hence, the entropy of the magnetic subsystem is lowered by the continuous energy transfer to the phonon subsystem, which, in turn, provides further stabilization of the ferromagnetic order.

In conclusion, we have considered ferromagnetism in the presence of chiral phonons. Contrary to the conventional perception of magnetic order, we have found that phonons play the role as an energy sink to which thermal energy provided by the environment may be disposed off, thereby preventing magnon excitations from becoming occupied and destroying the ferromagnetic order. A requirement for a viable coupling between the magnetic and vibrational degrees of freedom is (i) absence of inversion symmetry^6^ or (ii) non-trivial spin texture within the electronic structure to which the nuclear vibrations are coupled.^31^ The theoretical account provided here explains recent results of increasing coercivity with an increasing temperature observed for ribose aminooxazoline on a ferromagnetic substrate.^29^ A possible way to discern whether the proposed mechanism is viable would be to measure the molecular phonon spectrum via vibrational circular dichroism. Identifying the relevant modes that then can be selectively excited, using a circularly polarized infrared (IR) laser, should replicate the observed effect without requiring an increase in the temperature. By pumping the chiral lattice modes with the correct handedness, adjustable via a quarter-wave plate, the induced magnetization should be enhanced.

## References

[ref1] Keffer F. Handbuch der Physik; Springer-Verlag: New York, 1966.

[ref2] Ahmmad B.; Islam M. Z.; Billah A.; Basith M. A. Anomalous coercivity enhancement with temperature and tunable exchange bias in Gd and Ti co-doped BiFeO3 multiferroics. J. Phys. D: Appl. Phys. 2016, 49, 095001 10.1088/0022-3727/49/9/095001.

[ref3] Dhara B.; Tarafder K.; Jha P. K.; Panja S. N.; Nair S.; Oppeneer P. M.; Ballav N. Possible Room-Temperature Ferromagnetism in Self-Assembled Ensembles of Paramagnetic and Diamagnetic Molecular Semiconductors. J. Phys. Chem. Lett. 2016, 7, 4988–4995. 10.1021/acs.jpclett.6b02063. 27973877

[ref4] Mondal A. K.; Brown N.; Mishra S.; Makam P.; Wing D.; Gilead S.; Wiesenfeld Y.; Leitus G.; Shimon L. J. W.; Carmieli R.; Ehre D.; Kamieniarz G.; Fransson J.; Hod O.; Kronik L.; Gazit E.; Naaman R. Long-Range Spin-Selective Transport in Chiral Metal–Organic Crystals with Temperature-Activated Magnetization. ACS Nano 2020, 14, 16624–16633. 10.1021/acsnano.0c07569. 33095016 PMC7760088

[ref5] Fransson J. Vibrationally Induced Magnetism in Supramolecular Aggregates. J. Phys. Chem. Lett. 2023, 14, 2558–2564. 10.1021/acs.jpclett.3c00157. 36877808 PMC10026173

[ref6] Fransson J. Chiral phonon induced spin polarization. Phys. Rev. Res. 2023, 5, L022039 10.1103/PhysRevResearch.5.L022039.

[ref7] Fransson J. Temperature activated chiral induced spin selectivity. J. Chem. Phys. 2023, 159, 084115 10.1063/5.0155854. 37638628

[ref8] Fransson J.; Turin L. Current Induced Spin-Polarization in Chiral Molecules. J. Phys. Chem. Lett. 2024, 15, 6370–6374. 10.1021/acs.jpclett.4c01362. 38857512 PMC11194818

[ref9] Červenka J.; Katsnelson M. I.; Flipse C. F. J. Room-temperature ferromagnetism in graphite driven by two-dimensional networks of point defects. Nat. Phys. 2009, 5, 840–844. 10.1038/nphys1399.

[ref10] Bonilla M.; Kolekar S.; Ma Y.; Diaz H. C.; Kalappattil V.; Das R.; Eggers T.; Gutierrez H. R.; Phan M.-H.; Batzill M. Strong room-temperature ferromagnetism in VSe2 monolayers on van der Waals substrates. Nat. Nanotechnol. 2018, 13, 289–293. 10.1038/s41565-018-0063-9. 29459653

[ref11] Huang C.; Feng J.; Wu F.; Ahmed D.; Huang B.; Xiang H.; Deng K.; Kan E. Toward Intrinsic Room-Temperature Ferromagnetism in Two-Dimensional Semiconductors. J. Am. Chem. Soc. 2018, 140, 11519–11525. 10.1021/jacs.8b07879. 30130098

[ref12] Xu Q.; Schmidt H.; Zhou S.; Potzger K.; Helm M.; Hochmuth H.; Lorenz M.; Setzer A.; Esquinazi P.; Meinecke C.; Grundmann M. Room temperature ferromagnetism in ZnO films due to defects. Appl. Phys. Lett. 2008, 92, 082508 10.1063/1.2885730.

[ref13] Han S.-J.; Song J. W.; Yang C.-H.; Park S. H.; Park J.-H.; Jeong Y. H.; Rhie K. W. A key to room-temperature ferromagnetism in Fe-doped ZnO: Cu. Appl. Phys. Lett. 2002, 81, 4212–4214. 10.1063/1.1525885.

[ref14] Wang Y.; Huang Y.; Song Y.; Zhang X.; Ma Y.; Liang J.; Chen Y. Room-Temperature Ferromagnetism of Graphene. Nano Lett. 2009, 9, 220–224. 10.1021/nl802810g. 19072314

[ref15] Yu R.; Zhang W.; Zhang H.-J.; Zhang S.-C.; Dai X.; Fang Z. Quantized Anomalous Hall Effect in Magnetic Topological Insulators. Science 2010, 329, 61–64. 10.1126/science.1187485. 20522741

[ref16] Huang B.; Clark G.; Navarro-Moratalla E.; Klein D. R.; Cheng R.; Seyler K. L.; Zhong D.; Schmidgall E.; McGuire M. A.; Cobden D. H.; Yao W.; Xiao D.; Jarillo-Herrero P.; Xu X. Layer-dependent ferromagnetism in a van der Waals crystal down to the monolayer limit. Nature 2017, 546, 270–273. 10.1038/nature22391. 28593970

[ref17] Chang C.-Z. Marriage of topology and magnetism. Nat. Mater. 2020, 19, 484–485. 10.1038/s41563-020-0632-9. 32152560

[ref18] Bernevig B. A.; Felser C.; Beidenkopf H. Progress and prospects in magnetic topological materials. Nature 2022, 603, 41–51. 10.1038/s41586-021-04105-x. 35236973

[ref19] Rößler U. K.; Bogdanov A. N.; Pfleiderer C. Spontaneous skyrmion ground states in magnetic metals. Nature 2006, 442, 797–801. 10.1038/nature05056. 16915285

[ref20] Mühlbauer S.; Binz B.; Jonietz F.; Pfleiderer C.; Rosch A.; Neubauer A.; Georgii R.; Böni P. Skyrmion Lattice in a Chiral Magnet. Science 2009, 323, 915–919. 10.1126/science.1166767. 19213914

[ref21] Yu X. Z.; Onose Y.; Kanazawa N.; Park J. H.; Han J. H.; Matsui Y.; Nagaosa N.; Tokura Y. Real-space observation of a two-dimensional skyrmion crystal. Nature 2010, 465, 901–904. 10.1038/nature09124. 20559382

[ref22] Tokura Y.; Kanazawa N. Magnetic Skyrmion Materials. Chem. Rev. 2021, 121, 2857–2897. 10.1021/acs.chemrev.0c00297. 33164494

[ref23] Sang Y.; Tassinari F.; Santra K.; Zhang W.; Fontanesi C.; Bloom B. P.; Waldeck D. H.; Fransson J.; Naaman R. Chirality enhances oxygen reduction. Proc. Natl. Acad. Sci. U.S.A. 2022, 119, e2202650119 10.1073/pnas.2202650119. 35858429 PMC9335305

[ref24] Gupta A.; Sang Y.; Fontanesi C.; Turin L.; Naaman R. Effect of Anesthesia Gases on the Oxygen Reduction Reaction. J. Phys. Chem. Lett. 2023, 14, 1756–1761. 10.1021/acs.jpclett.2c03753. 36779610 PMC9940288

[ref25] Ozturk S. F.; Sasselov D. D. On the origins of life’s homochirality: Inducing enantiomeric excess with spin-polarized electrons. Proc. Natl. Acad. Sci. U.S.A. 2022, 119, e2204765119 10.1073/pnas.2204765119. 35787048 PMC9282223

[ref26] Ozturk S. F.; Liu Z.; Sutherland J. D.; Sasselov D. D. Origin of biological homochirality by crystallization of an RNA precursor on a magnetic surface. Sci. Adv. 2023, 9, eadg8274 10.1126/sciadv.adg8274. 37285423 PMC10246896

[ref27] Chou W.-Y.; Peng S.-K.; Chang F.-H.; Cheng H.-L.; Ruan J.-J.; Ho T.-Y. Ferromagnetism above Room Temperature in a Ni-Doped Organic-Based Magnetic Semiconductor. ACS Appl. Mater. Interfaces 2021, 13, 34962–34972. 10.1021/acsami.1c08967. 34269055

[ref28] Kondou K.; Shiga M.; Sakamoto S.; Inuzuka H.; Nihonyanagi A.; Araoka F.; Kobayashi M.; Miwa S.; Miyajima D.; Otani Y. Chirality-Induced Magnetoresistance Due to Thermally Driven Spin Polarization. J. Am. Chem. Soc. 2022, 144, 7302–7307. 10.1021/jacs.2c00496. 35414173 PMC9052755

[ref29] Kapon Y.; Brann L.; Yochelis S.; Fransson J.; Sasselov D. D.; Paltiel Y.; Ozturk S. F. Non-classical Temperature Dependence of Chirality-Induced Magnetization and Its Implications for RNA’s Homochirality. arXiv.org, e-Print Arch., Phys. 2024, arXiv:2412.05720 10.48550/arXiv.2412.05720.

[ref30] Ozturk S. F.; Bhowmick D. K.; Kapon Y.; Sang Y.; Kumar A.; Paltiel Y.; Naaman R.; Sasselov D. D. Chirality-induced avalanche magnetization of magnetite by an RNA precursor. Nat. Commun. 2023, 14, 6351 10.1038/s41467-023-42130-8. 37816811 PMC10564924

[ref31] Fransson J.; Thonig D.; Bessarab P. F.; Bhattacharjee S.; Hellsvik J.; Nordström L. Microscopic theory for coupled atomistic magnetization and lattice dynamics. Phys. Rev. Materials 2017, 1, 074404 10.1103/PhysRevMaterials.1.074404.

[ref32] Holstein T.; Primakoff H. Field Dependence of the Intrinsic Domain Magnetization of a Ferromagnet. Phys. Rev. 1940, 58, 1098–1113. 10.1103/PhysRev.58.1098.

